# The Other Side of Cocaine—The Bad Side

**Published:** 1886-09

**Authors:** A. W. Calhoun

**Affiliations:** Atlanta, Ga.


### THE OTHER SIDE OF COCAINE—THE BAD SIDE.

REMARKS OF A. W. CALHOUN, M. D., ATLANTA, GA., BEFORE THE MEDICAL ASSOCIATION OF GEORGIA AT ITS THIRTY-SEVENTH SESSION AT AUGUSTA, APRIL 23, 1886.

Reported for THE ATLANTA MEDICAL AND SURGICAL JOURNAL.

So much has been said of the good effects of cocaine and so little mention been made of any evil results following its use, I wish to call your attention to some of its bad effects, so far as my experience with it has taught me. I speak more particularly of its use in the treatment of eye diseases. In most of the operations upon the external portions of the ball, it gives almost perfect immunity from pain, and there is scarcely any operation upon the eye, in the making of which it does not give some relief from pain. Operations for strabismus, pterygium, foreign bodies in the cornea, chalazion, strictures of the tear-canal, iridectomy and soft cataract, are now almost universally made under its influence and with most marked success; but in the *extraction* of cataract lies the chief danger, in my opinion. Previous to its use, my average of success in cataract extractions was about 95 to 97 per cent. From the day I began using it to the day of discarding it in extractions, I lost more cases than in all my previous experience with cataract operations. To my mind the best evidence of the bad influence of the cocaine was the fact that immediately upon stopping its use in extractions, my formerly good average of results was achieved and has since been steadily maintained. The cause of failure in every instance was a want of proper union of the corneal wound, resulting in more or less extensive sloughing of the cornea, or a severe form of iritis with an outpouring of a large amount of lymph in the pupillary space and anterior chamber. Most frequently both of these results took place in the same case. Of course, this failure of the corneal wound to unite must have been brought about by the contact of the cocaine with the cut surfaces of the cornea. In each case every precaution was taken in regard to preparatory treatment, and also both during and subsequent to the operation. In most instances a four per cent. solution of the muriate cocaine with two per cent. boracic acid was used freely at first, but afterwards very sparingly, but in some instances only a two per cent. solution with the boracic acid was used. A solution of bichloride mercury (1 part to 10,000) was instilled into the conjunctival sac previous, during and after the operation, and all the instruments were dipped into the same fluid just before operating.

Why such results should take place in my hands and not in others I am unable to say. In only one other direction have I found the company which misery loves. The surgeons of the

Moorefield's Royal Ophthalmic Hospital of London have had a similar experience to my own, and their cases, as reported, present symptoms and results very like mine, viz., non-union of the wound, sloughing, etc.

To the oculist the introduction of cocaine is of the greatest help and assistance, enabling him to discard the use of chloroform almost altogether. I even make enucleations of the ball very nearly exclusively with the aid of cocaine, but it is only that most delicate of eye operations, cataract extraction, in which the danger lies. Our enthusiasm is very apt to keep our gaze fixed upon the bright side of things, and make us forgetful of the fact that there may be a shadowy side. If, by calling attention to what has been in my hands the bad side of cocaine, some over-enthusiastic brother can be made to realize the fact that he must be careful in the handling of this most valuable remedy, I will have accomplished my object.

